# On the Employment of Iodine Injections in Chronic Dysentery

**Published:** 1853-10

**Authors:** 


*On the employment of Iodine Injections in Chronic Dysentery.* By M. Delioux.—On considering the success which has crowned the practice of those practitioners who have ventured to apply the tincture of iodine to the surface of such delicate and irritable membranes as the pleura and peritoneum, it struck me that the endeavors to modify the lesions which give rise to diarrhœa and dysentery by this means would be equally successful. Iodine, although a powerful topical irritant, does not appear to me to be capable, especially when sufficiently diluted, of causing a more severe inflammation than other remedies, such as nitrate of silver, which may be injected with impunity into the great intestine. I prescribe the iodine injections in the following manner:—Alcoholic tincture of iodine, from 10 to 20 grammes (about 150 to 200 grains). Iodide of potassium, from 1 to 2 grammes (about 15 to 30 grains). Water, from 200 to 220 grammes (about 6 to 7 ounces). The iodine is thus maintained in solution by the alkaline iodide. I direct an emollient injection to be first administered, to clear out the intestine, and then the iodine injection, which thus acts at once, and with full force on the mucous membrane.

I commenced by experimenting with small doses of tincture of iodine; but after having assured myself of the innocuousness of this injection, I raised the dose by degrees, and found that the quantity might be increased without danger to 30 grammes (1 ounce and 12½ grains).

It often happens that the iodine injections cause only a slight colic; at other times, after the first or second, the alvine dejections augment, to diminish immediately, at the same time changing their character; at other times, they are diminished or suppressed immediately. In twelve cases reported in my "Memoirs" the intestinal affection was remarkably benefited or cured ten times; twice the treatment was unsuccessful, but did not aggravate the disease. To gaurd my patients against the possibility of suffering from the irritant effects of the iodine, I always at the same time prescribe a laudanum injection, which is to be administered in case the other should cause too much pain. In general, this last prescription does not require to be used; if it is necessary to have recourse to it, it is with thecertainty of remedying those effects, which with me have never been severe. I am of opinion that the iodine injections are also capable of acting on the surfaces of ulcers, abscesses, œdematous swellings, and hypertrophies of the cæcum and colon; an effect analogous to that produced by the application of tincture of iodine to the surfaces on the exterior of the body affected with ulceration, purulent sinuses, and insoluble tumors. Whether this agent acts by substitutive irritation, or by any other unexplained mode, it appears to me to exercise on the intestinal mucous membrane properties at once incarnative, healing, and resolvent. But it is not only on the superficies of the diseased organ that the injection is to act. The iodine is partly absorbed, and it is very probable that, consecutively, it may act on the thickened intestinal mucous membrane and mesenteric glands.—*New-York Journal of Medicine.*

